# Low coverage sequencing of three echinoderm genomes: the brittle star *Ophionereis fasciata*, the sea star *Patiriella regularis*, and the sea cucumber *Australostichopus mollis*

**DOI:** 10.1186/s13742-016-0125-6

**Published:** 2016-05-10

**Authors:** Kyle A. Long, Carlos W. Nossa, Mary A. Sewell, Nicholas H. Putnam, Joseph F. Ryan

**Affiliations:** Whitney Laboratory for Marine Bioscience, University of Florida, 9505 Ocean Shore Blvd., St Augustine, FL 32080 USA; Department of Ecology and Evolutionary Biology, Rice University, P.O. Box 1892, Houston, TX 77251-1892 USA; School of Biological Sciences, University of Auckland, Auckland, New Zealand; Department of Biology, University of Florida, Gainesville, FL 32611-8525 USA

**Keywords:** Echinoderms, Genome, Brittle star, Sea star, Sea cucumber

## Abstract

**Background:**

There are five major extant groups of Echinodermata: Crinoidea (feather stars and sea lillies), Ophiuroidea (brittle stars and basket stars), Asteroidea (sea stars), Echinoidea (sea urchins, sea biscuits, and sand dollars), and Holothuroidea (sea cucumbers). These animals are known for their pentaradial symmetry as adults, unique water vascular system, mutable collagenous tissues, and endoskeletons of high magnesium calcite. To our knowledge, the only echinoderm species with a genome sequence available to date is *Strongylocentrotus pupuratus* (Echinoidea). The availability of additional echinoderm genome sequences is crucial for understanding the biology of these animals.

**Findings:**

Here we present assembled draft genomes of the brittle star *Ophionereis fasciata*, the sea star *Patiriella regularis*, and the sea cucumber *Australostichopus mollis* from Illumina sequence data with coverages of 12.5x, 22.5x, and 21.4x, respectively.

**Conclusions:**

These data provide a resource for mining gene superfamilies, identifying non-coding RNAs, confirming gene losses, and designing experimental constructs. They will be important comparative resources for future genomic studies in echinoderms.

**Electronic supplementary material:**

The online version of this article (doi:10.1186/s13742-016-0125-6) contains supplementary material, which is available to authorized users.

## Data description

Echinodermata consists of five classes: Crinoidea (feather stars and sea lillies), Ophiuroidea (brittle stars and basket stars), Asteroidea (sea stars), Echinoidea (sea urchins, sea biscuits, and sand dollars), and Holothuroidea (sea cucumbers). These animals have a rich fossil record, unique biomechanical properties, experimentally tractable embryos, and as such have been a favorite subject of study for more than 150 years. Along with Hemichordata, Echinodermata form a clade called Ambulacraria that are the sister group of Chordata, which include vertebrates.

Echinoderms are easily recognized due to striking synapomorphies. The most obvious is their pentaradial (five-fold) body symmetry that is characteristic of adults (earlier stages exhibit bilateral symmetry). They have a unique water vascular system, which is characterized by canals connecting small tube feet on the lateral side of the animals. The water vascular system is used for essential functions such as feeding, locomotion, waste disposal, and respiration. The “spiny skin” from which these animals get their name is an endoskeleton made up of calcareous plates called ossicles that is composed of high magnesium calcite formed as solid test, plates or ossicles depending on the class of echinoderms. Lastly, the ossicles are connected by ligaments made of collagen that are normally rigid, but may become flexible upon various neuronal stimuli.

The vast majority of genomic work thus far has focused on the classic developmental model, the sea urchin *Stronglycentrotus purpuratus* [1]. The genome of this sea urchin has produced many important findings of great interest, including the discovery of a rearrangement event in evolution that led to an unusual Hox cluster organization [2], a well-characterized gene network for the specification of endoderm and mesoderm [3], and insight into the effect of ocean acidification on biomineralization [4]. More recently the genomes of the sea star *Patiria miniata*, the sea urchin *Lytechinus variegatus*, the sea cucumber *Parastichopus parvimensis*, and the brittle star *Ophiothrix spiculata* have been made available in Echinobase [5].

**Fig. 1:**
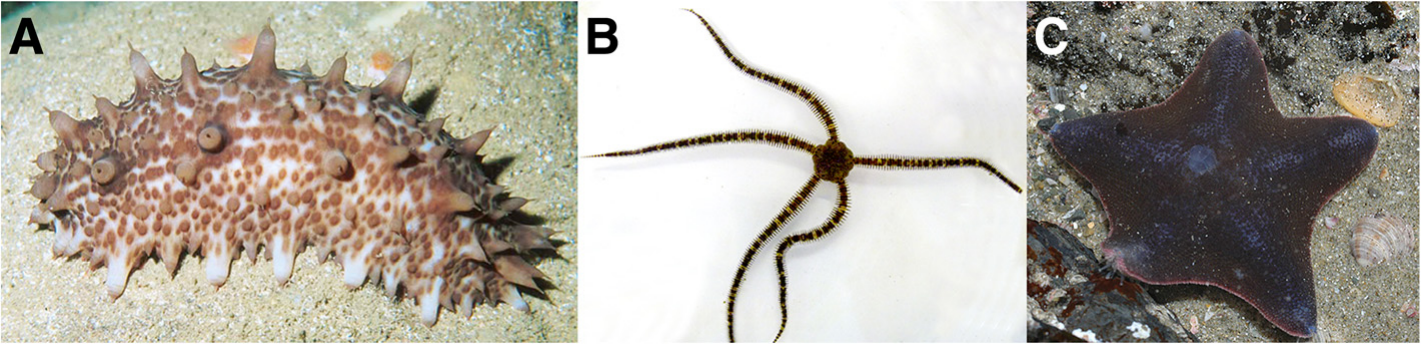
Photos of the echinoderm species whose genomes were sequenced in this study (**a**) *Australostichopus mollis*, commonly known as the brown sea cucumber; (**b**) the brittle star *Ophionereis fasciata* (**b**), and *Patiriella regularis*, known as the New Zealand common cushion star. Photo credits: (**a**) John A. Starmer, (**b**) Jennifer Howe, Victoria University of Wellington, and (**c**) username kiwi_kid on flickr (http://tinyurl.com/pregularis)

Here we provide genome sequences from species within three different echinoderm clades: *Australostichopus mollis*, commonly known as the brown sea cucumber (Fig. 1a), the brittle star *Ophionereis fasciata* (Fig. 1b), and *Patiriella regularis*, known as the New Zealand common cushion star (Fig. 1c). These species can be found in the shallow waters surrounding New Zealand.

These data can be used for the gene family phylogenetic analyses, domain/gene losses, and presence of small non-coding RNAs among other applications (e.g., [6]). They will be particularly useful in a comparative framework with existing Echinoderm genomes, for example to identify highly conserved non-coding regions. Finally, these data will be a key resource for labs working on these animals in the lab or in the field (e.g., designing markers, probes, and genome-editing constructs).

## Sample preparation and sequencing

Raw sequence data for all three species were produced from a single male, a single female, and the offspring of these two adults (Additional file 1: Table S1). For each individual sequenced, reads were barcoded and raw reads were submitted to the European Nucleotide Archive (ENA) separately (Additional file 2: Table S2). This sequencing strategy was originally used in order to employ a genetic mapping approach to genome assembly [7], and is not ideal for the standard *de novo* assembly strategy described herein. Nevertheless, when it became apparent that genetic mapping approach would not be feasible due to personnel, we applied a more traditional approach, and generated a useful set of data.

Parental tissue and larvae from each species were stored in ethanol prior to DNA extraction. DNA was extracted using Zymo DNA clean kit and was used to prepare Nextera libraries for 2 × 100 PE (paired end) sequencing on Illumina HiSeq2000 at BGI Shenzhen.

## Assembly

For all steps of the assembly, all reads from each species were pooled (i.e., 2 parents and offspring). We used ALLPATHS-LG v44837 [8] to correct errors in the raw reads. We used Cutadapt v1.4.2 [9] to remove adapter sequences. We assembled these data using three assemblers: SOAPdenovo2 r2.04 [10], ABySS 3.81 [11], and Platanus 1.1.2 [12]. For each program we used a range of k-mer values: 31, 39, 45, 55, 63 for SOAP and ABySS, and 32, 39, 45, 55, 64 for Platanus. This provided a total of 15 assemblies for each organism. Each assembly/k-mer combination was evaluated using N50 values and number of conserved eukaryotic genes recovered by CEGMA 2.4 [13]. In all cases, SOAPdenovo assemblies were deemed superior to those produced by ABySS and Platanus (Table 1). The sequences represent coverages of 12.5x, 22.5x, and 21.4x for the brittle star, sea star, and sea cucumber, respectively.

**Table 1:** N50 values and CEGMA scores for each Assembly

Species	Assembler	K-mer Value	N50 (bp)	# Contigs	CEGMA Score (Partial/Complete)
*O. fasciata*	**SOAPdenovo**	31	484	3,968,282	69/16
		39	451	4,740,140	69/17
		**45**	**501**	**4,814,066**	**71/20**
		55	449	5,961,782	63/9
		63	528	4,184,863	57/5
	ABySS	31	70	32,912,859	34/6
		39	99	24,109,265	32/1
		45	116	19,642,981	26/1
		55	146	18,334,263	0/0
		63	166	13,153,732	0/0
	Platanus	32	103	126	0/0
		39	104	129	0/0
		45	108	119	0/0
		55	105	130	0/0
		64	111	119	0/0
*P. regularis*	**SOAPdenovo**	31	1383	19,728	1/0
		39	469	2,348,237	17/1
		45	488	2,596,707	15/0
		55	470	3,424,052	22/1
		**63**	**557**	**3,006,458**	**50/2**
	ABySS	31	70	14,653,947	12/1
		39	96	11,632,456	14/2
		45	116	9,818,773	15/3
		55	158	6,986,935	22/3
		63	191	5,068,075	19/4
	Platanus	32	155	1,923,405	0/0
		39	157	2,475,110	0/0
		45	161	2,814,106	0/0
		55	158	3,044,990	0/0
		64	143	3,092,862	0/0
*A. mollis*	**SOAPdenovo**	31	847	2,132,880	77/16
		39	564	3,162,372	62/12
		45	577	3,457,710	66/13
		55	475	4,839,379	66/17
		**63**	**626**	**3,712,641**	**87/18**
	ABySS	31	76	19,911,844	44/5
		39	104	15,084,417	48/5
		45	127	12,518,443	52/7
		55	171	8,798,225	42/5
		63	204	6,232,955	32/0
	Platanus	32	214	2,653,880	0/0
		39	182	3,763,387	0/0
		45	164	4,653,306	0/0
		55	104	102	0/0
		64	106	99	0/0

Assemblies deemed to be the best for each species based on N50 and CEGMA scores are in bold and were used for all downstream analyses

## Gene prediction

We used Augustus v3.0.3 [14] to generate *ab initio* gene predictions for the best assemblies of each of the echinoderm species. We created a training set with the *Strongylocentrotus purpuratus* v3.1 scaffolds and corresponding predicted gene models from Echinobase [5]. We used “generic,” “human,” and the custom model “strongylocentrotus_purpuratus” as values for the -species parameter. To compare the three different sets of gene predictions we BLASTed both human and *S. purpuratus* protein models against the protein predictions from Augustus using BLASTP v2.2.31+ [15] with an E-value cutoff of 1e-6 and limiting to a single target sequence (blastp -query query.fa -db aug.fa -evalue 1e-6 -outfmt 6 -max_target_seqs 1). For all species BLAST searches against the Augustus protein sequences generated with the custom *S. purpuratus* model resulted in the most hits (Additional file 3: Table S3). We therefore chose these predictions as our final sets.

We generated 49,301 *A. mollis*, 102,838 *O. fasciata*, and 1135 *P. regularis* gene predictions (Additional file 4: Table S4). In the case of *A. mollis* and *O. casciata* these numbers are substantially higher than the 22,709 published *S. purpuratus* gene models. The high number in *A. mollis* and *O. casciata* might be due to multiple fragmented predictions representing single genes. *P. regularis* has substantially fewer predictions, which might suggest that in most cases, the scaffolds were too short even to predict partial genes.

## Data availability

All data including sequencing reads and assemblies have been submitted to the ENA under the following project accessions: *Australostichopus mollis* = PRJEB10682; *Patiriella regularis* = PRJEB10600; *Ophionereis fasciata* = PRJEB10339. Supporting data is also archived in the *GigaScience* GigaDB database [16], and additional resources are available from http://ryanlab.whitney.ufl.edu/genomes/.

## Additional files

Additional file 1: Table S1. Animal collection details (DOCX 23 kb) Additional file 2: Table S2. Sequencing sample details (DOCX 24 kb) Additional file 3: Table S3. BLASTP results of human RefSeq proteins (20,379 sequences) and *Strongylocentrotus purpuratus* proteins (22,709 sequences) against Augustus predictions run with generic, human, and *Strongylocentrotus purpuratus* training sets (DOCX 23 kb) Additional file 4: Table S4. Number of gene models and N50 values from Augustus predictions run with generic, human, and *Strongylocentrotus purpuratus* training sets (DOCX 23 kb)

## Abbreviations

ENA: European Nucleotide Archive
DNA: deoxyribonucleic acid
PE: paired end
RNA: ribonucleic acid
