# Supplementary material for: Low coverage sequencing of three echinoderm genomes: the brittle star Ophionereis fasciata, the sea star Patiriella regularis, and the sea cucumber Australostichopus mollis
Source: Gigascience. 2016 May 10;5:20. doi: 10.1186/s13742-016-0125-6 (PMC4863316; doi:10.1186/s13742-016-0125-6)
Supplement: Additional file 1: Table S1. — Animal collection details (DOCX 23 kb) [file 13742_2016_125_MOESM1_ESM.docx]

**Additional file 1: Table S1 - Animal collection details**

| Species | Geography | Depth | Date | Collected | Cultures | Spawn |
| --- | --- | --- | --- | --- | --- | --- |
| *Patiriella regularis* | Kohimarama Beach, New Zealand  36^o^50’50.83S  174^o^50’17.57E | intertidal | 13 Jan 2013 | Walk | 14 Jan 2013 | Dissected gonads into 16 µM 1-MA |
| *Ophionereis fasciata* | Matheson’s Bay, New Zealand  36^o^18’05.41S  174^o^48’01.69E | 1.5-2 m | 20 Jan 2013 | Snorkel | 21 Jan 2013 | Capture stress |
| *Australostichopus mollis* | Ti Point, New Zealand  36º 19’20.39” S; 174º47’28.7” E) | 4-15 m | 29 Jan 2013 | SCUBA | 12 Feb 2013 | Temperature induction |
