# Supplementary material for: Low coverage sequencing of three echinoderm genomes: the brittle star Ophionereis fasciata, the sea star Patiriella regularis, and the sea cucumber Australostichopus mollis
Source: Gigascience. 2016 May 10;5:20. doi: 10.1186/s13742-016-0125-6 (PMC4863316; doi:10.1186/s13742-016-0125-6)
Supplement: Additional file 2: Table S2. — Sequencing sample details (DOCX 24 kb) [file 13742_2016_125_MOESM2_ESM.docx]

**Additional file 2: Table S2 – Sequencing sample details**

| **Species/Study** | **ENA Accession** | **Sample Name** | **Life Stage** |
| --- | --- | --- | --- |
| *Patiriella regularis*  (PRJEB10600) | ERX1074423 | Preg_larvae_01 | larval |
|  | ERX1074424 | Preg_larvae_02 | larval |
|  | ERX1074425 | Preg_larvae_03 | larval |
|  | ERX1074426 | Preg_larvae_04 | larval |
|  | ERX1074427 | Preg_larvae_05 | larval |
|  | ERX1074428 | Preg_larvae_06 | larval |
|  | ERX1074429 | Preg_larvae_07 | larval |
|  | ERX1074430 | Preg_larvae_08 | larval |
|  | ERX1074431 | Preg_larvae_09 | larval |
|  | ERX1074432 | Preg_larvae_10 | larval |
|  | ERX1074433 | Preg_larvae_11 | larval |
|  | ERX1074434 | Preg_larvae_12 | larval |
|  | ERX1074435 | Preg_larvae_13 | larval |
|  | ERX1074436 | Preg_larvae_14 | larval |
|  | ERX1074437 | Preg_larvae_15 | larval |
|  | ERX1074438 | Preg_larvae_16 | larval |
|  | ERX1074439 | Preg_larvae_17 | larval |
|  | ERX1074440 | Preg_larvae_18 | larval |
|  | ERX1074441 | Preg_larvae_19 | larval |
|  | ERX1074442 | Preg_larvae_20 | larval |
|  | ERX1074443 | Preg_larvae_21 | larval |
|  | ERX1074444 | Preg_parent_female | adult (female) |
|  | ERX1074445 | Preg_parent_male | adult (male) |
| *Ophionereis fasciata*  (PRJEB10339) | ERX1066686 | Ofas_larva_01 | larval |
|  | ERX1066687 | Ofas_larva_02 | larval |
|  | ERX1066688 | Ofas_larva_03 | larval |
|  | ERX1066689 | Ofas_larva_04 | larval |
|  | ERX1066690 | Ofas_larva_05 | larval |
|  | ERX1066691 | Ofas_larva_06 | larval |
|  | ERX1066692 | Ofas_larva_07 | larval |
|  | ERX1066693 | Ofas_larva_08 | larval |
|  | ERX1066694 | Ofas_larva_09 | larval |
|  | ERX1066695 | Ofas_larva_10 | larval |
|  | ERX1066696 | Ofas_larva_11 | larval |
|  | ERX1066697 | Ofas_larva_12 | larval |
|  | ERX1066698 | Ofas_larva_13 | larval |
|  | ERX1066699 | Ofas_larva_14 | larval |
|  | ERX1066700 | Ofas_larva_15 | larval |
|  | ERX1066701 | Ofas_larva_16 | larval |
|  | ERX1066702 | Ofas_larva_17 | larval |
|  | ERX1066703 | Ofas_larva_18 | larval |
|  | ERX1066704 | Ofas_larva_19 | larval |
|  | ERX1066705 | Ofas_larva_20 | larval |
|  | ERX1066706 | Ofas_larva_21 | larval |
|  | ERX1066707 | Ofas_larva_22 | Larval |
|  | ERX1066708 | Ofas_parent_1 | adult (sex?) |
|  | ERX1066709 | Ofas_parent_2 | adult (sex?) |
| *Australostichopus mollis*  (PRJEB10682) | ERX1080679 | Amol_larvae_01 | larval |
|  | ERX1080680 | Amol_larvae_02 | larval |
|  | ERX1080681 | Amol_larvae_03 | larval |
|  | ERX1080682 | Amol_larvae_04 | larval |
|  | ERX1080683 | Amol_larvae_05 | larval |
|  | ERX1080684 | Amol_larvae_06 | larval |
|  | ERX1080685 | Amol_larvae_07 | larval |
|  | ERX1080686 | Amol_larvae_08 | larval |
|  | ERX1080687 | Amol_larvae_09 | larval |
|  | ERX1080688 | Amol_larvae_10 | larval |
|  | ERX1080689 | Amol_larvae_11 | larval |
|  | ERX1080690 | Amol_larvae_12 | larval |
|  | ERX1080691 | Amol_larvae_13 | larval |
|  | ERX1080692 | Amol_larvae_14 | larval |
|  | ERX1080693 | Amol_larvae_15 | larval |
|  | ERX1080694 | Amol_larvae_16 | larval |
|  | ERX1080695 | Amol_larvae_17 | larval |
|  | ERX1080696 | Amol_larvae_18 | larval |
|  | ERX1080697 | Amol_larvae_19 | larval |
|  | ERX1080698 | Amol_larvae_20 | larval |
|  | ERX1080699 | Amol_larvae_21 | larval |
|  | ERX1080700 | Amol_larvae_22 | Larval |
|  | ERX1080701 | Amol_parent_01 | adult (sex?) |
|  | ERX1080702 | Amol_parent_02 | adult (sex?) |
