# Supplementary material for: Low coverage sequencing of three echinoderm genomes: the brittle star Ophionereis fasciata, the sea star Patiriella regularis, and the sea cucumber Australostichopus mollis
Source: Gigascience. 2016 May 10;5:20. doi: 10.1186/s13742-016-0125-6 (PMC4863316; doi:10.1186/s13742-016-0125-6)
Supplement: Additional file 3: Table S3. — BLASTP results of human RefSeq proteins (20,379 sequences) and Strongylocentrotus purpuratus proteins (22,709 sequences) against Augustus predictions run with generic, human, and Strongylocentrotus purpuratus training sets (DOCX 23 kb) [file 13742_2016_125_MOESM3_ESM.docx]

**Additional file 3: Table S3 – BLASTP results of human RefSeq proteins (20,379 sequences) and *Strongylocentrotus purpuratus* proteins (22,709 sequences) against Augustus predictions run with generic, human, and *Strongylocentrotus purpuratus* training sets**

| **Augustus parameter** | --species=generic | | --species=human | | --species=strongylo | |
| --- | --- | --- | --- | --- | --- | --- |
| **BLASTP query** | HumRef | *S.pur* | HumRef | *S.pur* | HumRef | *S.pur* |
| *Patiriella regularis* | 1,719 | 2,895 | 2,289 | 3,655 | 2,616 | 4,213 |
| *Ophionereis fasciata* | 10,699 | 15,107 | 11,057 | 15,364 | 13,246 | 18,839 |
| *Australostichopus mollis* | 8,489 | 12,083 | 10,614 | 14,623 | 11,784 | 16,573 |
