# Supplementary material for: Low coverage sequencing of three echinoderm genomes: the brittle star Ophionereis fasciata, the sea star Patiriella regularis, and the sea cucumber Australostichopus mollis
Source: Gigascience. 2016 May 10;5:20. doi: 10.1186/s13742-016-0125-6 (PMC4863316; doi:10.1186/s13742-016-0125-6)
Supplement: Additional file 4: Table S4. — Number of gene models and N50 values from Augustus predictions run with generic, human, and Strongylocentrotus purpuratus training sets (DOCX 23 kb) [file 13742_2016_125_MOESM4_ESM.docx]

**Additional file 4: Table S4 – Number of gene models and N50 values from Augustus predictions run with generic, human, and *Strongylocentrotus purpuratus* training sets**

| **Augustus parameter** | --species=generic | | --species=human | | --species=strongylo | |
| --- | --- | --- | --- | --- | --- | --- |
|  | # models | N50 | # models | N50 | # models | N50 |
| *Patiriella regularis* | 491 | 250 aa | 516 | 105 aa | 1,135 | 188 aa |
| *Ophionereis fasciata* | 52,670 | 177 aa | 43,823 | 197 aa | 102,838 | 121 aa |
| *Australostichopus mollis* | 21,275 | 199 aa | 23,092 | 223 aa | 49,301 | 131 aa |
